# 

**Published:** 1901-08-10

**Authors:** 


### NOTICE TO CORRESPONDENTS.

All MSS., Letters, Books for Review, and other matters intended for the Editor should be addressed The Editor, "The Hospital," The Hospital Building, 28 & 29 Southampton Street, Strand, W.C.

The Editor cannot undertake to return rejected MS., even when accompanied by stamped directed envelope.

**Notice to Advertisers and Subscribers.**

All Advertisements, Orders for Copies of The Hospital, and Business Communications should be addressed to **THE MANAGER (Not to the Editor),** The Hospital, 28 & 29 Southampton Street, Strand, London, W.C.

The Cost of Subscription, post free, is as follows:—Per week, 2½d.; per quarter, 2s. 8d.; per half-year, 5s. 3d.; per annum, 10s. 6d. Foreign:—Per annum, 15s. 2d., payable, in all cases, in advance.

**NOTICE.—Vol. XXIX. of "The Hospital" (October 1900. to March, 1901), in handsome cloth binding is now on sale at the Office of this Journal, price, 7s. 6d. Cases for binding the Numbers contained in this Volume 1s. 6d. Index to Vol. XXIX., 2½d., post free.**

**The Monthly Part for August is now ready. Price 6d., by post 8d.**

### BOOKS RECEIVED.

G. Cornwall and Sons.

"Summer Trips to Norway," etc.

Cassell and Co.

"Cassell's Family Doctor." By a Medical Man.

"Surgical Diseases of the Kidney and Ureter." By Henry Morris, M.A., M.B.Lond., F.R.C.S. In two volumes.

West Sussex Gazette Office.

"Vexed Questions." By Rachel Challice.

John Wright and Co.

"Our Baby." By Mrs. Langton Hewer.

George Bell and Sons.

"The Penalty of Death." By Josiah Oldfield, M.A., B.C.L.(Oxon.) L.R.C.P., M.R.C.S.

### Scraps and Gleanings.

The estimated cost of the new Victoria Cottage Hospital at Southwold is £2,000: it provides ten beds on the ground floor and five bedrooms on the second floor.

After some discussion by the Canterbury Town Council it was decided to adopt the report of the Sanatorium Committee, who recommend that additional accommodation shall be provided for the nursing staff of the sanatorium, and that application be made to the Local Government Board for sanction to borrow £600 in order to carry out these works.

At a recent extraordinary general meeting of the Royal Albert Edward Infirmary, Wigan, it was resolved that ladies should be appointed visitors of the Infirmary as well as men. At the annual general meeting of the institution held immediately after this extraordinary meeting the chairman of the board of management stated that they would have to look forward to the establishment of a home for the nursing staff. The new isolation block which had been built for infectious diseases would soon be ready for opening, and it was necessary in the near future to overhaul the flooring of the older wards and to retile the vestibule and corridors.

### The Student of Medicine.

### Appointments.

**W. T. D. Allen**, M.B., B.Ch., R.U.I., appointed Medical Officer for the 4th District of the Parish of Liverpool. **E. W. Barnes**, L.R.C.P.I., L.F.P.S.Glasg., appointed District Medical Officer to the Liverpool Union. **A. H. Buck**, F.R.C.S., M.R.C.S., L.R.C.P., appointed Honorary Surgeon to the Brighton, Hove, and Sussex Throat and Ear Hospital. **Arthur H. Burgess**, F.R.C.S.Eng., M.B., Ch.B., M.Sc.Vict., appointed Surgical Officer and Medical Superintendent of the Manchester Cancer Pavilion and Home. **H. Hollis**, M.D., appointed Medical Officer for the Wellingborough District and the Workhouse of the Wellingborough Union also Vaccination Officer. **A. J. Hutchison**, M.A., M.B., C.M., appointed Honorary Assistant-Surgeon to the Brighton, Hove, and Sussex Throat and Ear Hospital. **Alice Vowe Johnson**, L.R.C.P.&S.Edin., L.S.A.Lond., M.D.Brux., appointed Junior Medical Officer to the Joint Counties Asylum, Carmarthen. **Helen MacMurchy**, M.D.Toronto, appointed a Resident Medical Assistant to the General Hospital, Toronto, Canada. **A. J. Martineau**, F.R.C.S., M.R.C.S., L.R.C.P., appointed Honorary Assistant-Surgeon to the Brighton, Hove, and Sussex Throat and Ear Hospital. **J. W. Meade**, L.R.C.P., L.R.C.S.E., appointed Assistant Resident Surgeon to Nottingham General Dispensary. **George A. Rorie**, M.B., Ch.B., M.P.C., appointed Senior Assistant, Dorset County Asylum, Dorchester. **Miss A. B. Sinclair**, M.B., Ch.B., appointed Resident House-Surgeon to the Carlisle Dispensary. **T. Rudolph Smith**, M.B., F.R.C.S., appointed Honorary Surgeon to the Stockton and Thornaby Hospital. **J. Godfrey C. Taunton**, M.D., M.R.C.S.Eng., L.R.C.P.Lond., appointed Honorary Surgeon to St. Bartholomew's Hospital, Rochester. **St. Clair Thomson**, M.D., M.R.C.P.Lond., F.R.C.S.Eng., appointed Assistant Physician for Diseases of the Throat to King's College Hospital. **Edward Treves**, M.R.C.S., L.R.C.P., appointed Honorary Consulting Surgeon to the Brighton, Hove, and Sussex Throat and Ear Hospital. **Wilfrid B. Warde**, M.D., M.R.C.P., appointed Assistant-Physician to the Hospital for Diseases of the Skin, Blackfriars, London.

